# Antibiotic prophylaxis in reduction mammaplasty: study protocol for a randomized controlled trial

**DOI:** 10.1186/s13063-016-1700-y

**Published:** 2016-11-30

**Authors:** Edgard Silva Garcia, Daniela Francescato Veiga, Joel Veiga-Filho, Isaías Vieira Cabral, Natália Lana Larcher Pinto, Neil Ferreira Novo, Miguel Sabino Neto, Lydia Masako Ferreira

**Affiliations:** 1 Translational Surgery Graduate Program, Federal University of São Paulo, Rua Napoleão de Barros, 725, 4th Floor, Vila Clementino, CEP: 04024-002 São Paulo, SP Brazil; 2 Division of Plastic Surgery, Universidade do Vale do Sapucaí, Rua Comendador José Garcia, 777, Centro, CEP: 37550-000 Pouso Alegre, MG Brazil

**Keywords:** Plastic surgery, Mammaplasty, Wound infection, Prophylaxis

## Abstract

**Background:**

The role of antibiotics in surgical procedures where the risk of surgical site infection (SSI) is low remains uncertain. There is, to date, no evidence to justify the routine use of antibiotics in postoperative reduction mammaplasty. The aim of this study is to evaluate the effect of postoperative antibiotic treatment on the occurrence of SSI after breast reduction surgery.

**Methods:**

This is a double-blind randomized clinical trial with 124 breast hypertrophy patients allocated to two treatment groups: antibiotic (*n* = 62) and placebo (*n* = 62). All patients will undergo reduction mammoplasty, performed by the same surgical team. The surgeons will raise the nipple-areola complex by the superomedial pedicle technique. The patients will receive antibiotics intravenously during anesthetic induction and every 6 hours thereafter during their 24-hour hospital stay. During discharge from the hospital, each patient will receive a numbered package containing either cephalexin or placebo capsules and will be directed to take one capsule every 6 hours for 7 days. Neither the surgery team nor the patients will know the contents of the capsules. Patients will be monitored for the occurrence of SSI once weekly during the first 30 days following hospital discharge by a single surgeon who is blinded to their treatment group. SSI will be evaluated based on the definition adopted by the Centers for Disease Control and Prevention.

**Discussion:**

Due to the variety of risk factors for SSI and limited case studies, conclusions regarding the effect of antibiotics on the occurrence of SSIs following reduction mammaplasty are potentially biased. In recent studies, perioperative antibiotic prophylaxis was effective in preventing infection and is therefore recommended in clinical practice. However, antibiotic use in the postoperative period still remains controversial.

**Trial registration:**

Clinicaltrials.gov Identifier: NCT02569866. Registered on 4 October 2015.

## Background

Surgical site infections (SSIs) are defined as wound infections that occur after invasive procedures and remain among the most common infections, accounting for 14–16% of all nosocomial infections in hospitalized patients. In recent years, greater attention has been paid to the study, prevention and control of SSIs by surgeons, health care professionals, and patients [1–4].

In a literature review of potential factors affecting the occurrence of SSIs, Junker et al. noted that the timing of antibiotic administration and the occurrence of surgical glove perforation significantly influence the occurrence of SSIs. Other factors, such as patient anemia, blood transfusion during surgery, and surgeon experience were not significant [3].

In many surgical procedures where the SSI risk is low, the role of antibiotics remains uncertain. Despite the lack of evidence of efficacy from randomized controlled trials, its use in plastic surgery is widespread in order to offer patients greater presumed safety standards, because even minor infections are able to interfere with the healing process and therefore the aesthetic result [4–11].

The prophylactic use of antibiotics was first studied by Krizek et al., in 1975. These authors evaluated the prescribing habits of surgeons over 10 years and demonstrated an increased use of prophylaxis during this period [3, 5, 12–15]. In a recent review article, Sajid et al. concluded that prophylactic antibiotic therapy reduces the risk of SSI after breast surgery and the risk of adverse reactions to this therapy was not significant [16].

SSIs related to breast surgeries occur at rates ranging from 0.8 to 36%, which is the highest among clean surgeries as a whole (rate lower than 5%). This variation can be attributed to the type of breast surgery performed, patient comorbidities, duration of postoperative follow-up, intraoperative complications and the availability of institutional information [17–19]. Nevertheless, there is, to date, no evidence to justify the routine use of antibiotics in the postoperative of reduction mammaplasty, and there is no consensus regarding its prescription, even among surgeons of the same department [14, 20–22].

The Centers for Disease Control and Prevention (CDC) define the specific criteria for the diagnosis of SSIs as purulent drainage, positive aseptic culture, or signs or symptoms of inflammation upon opening the incision beyond superficial cellulitis [2, 19].

The impact of infection on wound healing after breast reduction surgery ranges from minor complications, such as marginal necrosis, which is common at the junction of the inverted “T” incision, to major infections that require surgical intervention [23]. Many surgeons prefer to administer antibiotics, believing that it will reduce the incidence of these problems; however, previous studies suggest that antibiotic prophylaxis fails to reduce the rates of infection in clean surgeries, such as breast reduction [7, 14, 23–26].

Among studies conducted in different countries (United Kingdom, Ireland, Israel, and the United States), there was a tendency to prescribe prophylactic antibiotics beyond the intra- and postoperative periods (24 hours) of breast reduction, ranging up to 14 days. Cephalosporins were the most frequently prescribed, with the exception of the UK, which mentioned the use of amoxicillin/clavulanic acid in 70% of cases [9, 19, 21–23, 27–31].

Veiga-Filho et al. [32] compared SSI rates in patients receiving cephalexin for 7 days after hospital discharge (24 hours after breast reduction surgery) to patients who did not receive antibiotics. They observed significantly higher SSI incidence rates in the control group compared with the experimental group receiving antibiotics (14% vs. 2%, respectively; *p* = 0.03). They also observed that the incidence of SSIs was greater in older patients and in patients with a greater resected breast tissue mass (*p* = 0.02; *p* = 0.04, respectively) [32].

Reduction mammaplasty is an effective procedure that improves the quality of life for women with breast hypertrophy and is one of the most common plastic surgery procedures [33–36]. Prevention of SSIs is extremely important, because infection may compromise the outcome of the procedure, and increase the length of stay and costs of hospitalization [37].

This study was designed to address the lack of consensus on the role of antibiotics in breast reduction surgery. Despite the absence of solid evidence, the use of antibiotics following reduction mammaplasty is widespread, and a previous nonrandomized trial demonstrated higher SSI rates when no antibiotics were used. Thus, this trial was designed to compare the effectiveness of antibiotics prophylaxis for 24 hours with the extended use of antibiotics for 7 additional days.

The objective of this study is to evaluate the effect of postoperative antibiotic treatment on SSI prevention in breast reduction with perioperative prophylaxis.

## Methods/Design

This is a randomized clinical trial with two parallel, double-blind groups.

### Study setting

This single-center study is being conducted in the Academical Hospital of the Universidade do Vale do Sapucaí. The hospital is located in the urban area of Pouso Alegre, Minas Gerais, Brazil.

### Eligibility criteria

Patients who meet the eligibility criteria and provide their consent by signing an informed consent form are eligible to join the study.

#### Inclusion criteria

Female patients are included in this study if they are between 18 and 60 years of age, have a body mass index (BMI) between 19 and 30 kg/m^2^ and have breast hypertrophy according to the criteria of Sacchini ﻿et al. [38] and Franco and Rebello [39].

#### Exclusion criteria

Patients are excluded if they had previously undergone another surgical procedure of the breast, have been diagnosed with a breast pathology, are former smokers, have had a child or breastfed within the last year, have uncontrolled comorbidities, such as arterial hypertension or diabetes, or take immunosuppressants. Further, patients who misuse capsules or miss follow-up assessments will be excluded from analysis.

## Interventions

### Surgical procedures

The patients will undergo reduction mammaplasty in the surgical center of the hospital of the Universidade do Vale do Sapucaí, by the same surgical team, lead by a single surgeon. The superomedial pedicle technique will be used to raise the nipple-areola complex [40].

All patients will be hospitalized the day before surgery and will take a shower with a 4% chlorhexidine antiseptic solution on the day of surgery [41, 42]. The operation will be performed under general anesthesia, and antisepsis of the operative field will be carried out with a 0.5% chlorhexidine alcohol solution [41, 42]. All patients will receive antibiotics intravenously during surgery and during their hospital stay (cephalothin, 1 g at induction of anesthesia and every 6 hours).

### Outcome procedures

On the first postoperative day, prior to hospital discharge, the assistant surgeon will remove the dressing from the incision and will instruct the patient on how to wash the wound daily with soap and water. Patients will also be instructed to wear a postoperative bra (MaCom® - Malhas de Compressão, Brazil - Ref 1002) for 30 days, and to avoid driving and working out for the same period.

After 24 hours, at hospital discharge, the assistant surgeon will give the patient a package with her protocol number, containing 28 capsules (cephalexin 500 g or placebo 500 g), and instruct her to take one every 6 hours for 7 days. Specially produced capsules containing either the antibiotic or placebo were kindly provided by Cimed Indústria de Medicamentos Ltda, São Paulo, Brrazil, who was not involved with design or conduct of this study in any other way.

### Adherence

#### Adherence reminder sessions

Face-to-face reminder sessions as to the importance of treatment adherence occur the day before surgery and at each weekly postoperative appointment. Such sessions include:

- Clear instructions are provided for all prescriptions and, after asking whether the patient has any questions, the patient is asked to repeat the instructions in their own words.
- The confirmation about the correct use of the capsules during the first 7 days of the postoperative period.
- The importance of following medical recommendations on the use of the bra and surgical wound care.
- The questioning of possible events that could constitute exclusion criteria as described in methods.

### Outcome measures

The CDC considers an infection to be an SSI when it appears to be related to the surgical procedure and presents within 30 days of the operation. If implants are inserted during the surgery, the time frame is extended up to a year after the surgery [1]. CDC classifies SSI into three categories, superficial incisional, deep incisional or organ/space SSI. In the present study, as none of the patients received an implant, requiring a delayed assessment, patients will be monitored for the occurrence of infection once a week for the first 30 days following surgery as assessed by a single surgeon, who is a senior plastic surgeon, with extensive experience in breast surgery. This surgeon will diagnose the presence or absence of SSI, thus dichotomizing the outcome as development of SSI (Yes/No). In case of a SSI, it will be classified according to CDC’s criteria, by the same surgeon [1].

### Sample size

The experimental and control groups were designed for 62 patients each to ensure a significance level of 5% and power of 80%. This was calculated based on the proportions observed in a previous study with and without the use of antibiotics after breast reduction [32].

### Recruitment

The recruitment started in April 2012. The 124 breast hypertrophy patients will be selected from the plastic surgery clinics of the hospital of the Universidade do Vale do Sapucaí, where these procedures are routinely performed and followed, ensuring patient compliance. All surgeries will be performed in the operating room of this hospital.

### Allocation – randomization

Patients will be randomly allocated into the study groups based on a random sequence generated by Bioestat 5.0 software (Instituto Mamirauá, Belém, Brazil). Patients in the experimental group (*n* = 62) will receive 500 mg cephalexin capsules, while patients in the placebo group (*n* = 62) will receive placebo capsules, that are identical in appearance to the antibiotic capsules. The antibiotic and placebo capsules were provided in identical packages containing the exact number of capsules for each patient.

### Allocation – concealment mechanism

A researcher, who will not participate in the care of patients, numbered the packages from 1 to 124 as per the randomly generated sequence. At the time of hospital discharge, each patient will receive a serial number and the corresponding numbered package containing the capsules that she will take.

### Allocation – implementation

Only one of the researchers will have access to the random sequence. He will not be responsible for recruiting patients, performing surgical procedures, distributing the packages, or performing postoperative follow-up, ensuring allocation concealment.

### Blinding

The surgery team, the assistant surgeon who instructs patients regarding the capsule intake and the surgeon who evaluates the SSIs are blinded to the allocation group. In addition, the patients are not informed about the contents of their prescribed capsules.

The first assessment regarding SSI is after 7 days. However, patients are instructed to contact the assistant surgeon in case of pain, fever or any other intercurrence before this first assessment. If the assistant surgeon diagnoses a SSI during the use of the antibiotic or placebo, the appropriate antibiotic treatment is immediately established and the use of the study capsules is suspended, without unblinding. Table 1 presents the schedule of the trial.

**Table 1:** Schedule of enrolment, interventions, and assessments for “Antibiotic prophylaxis in reduction mammaplasty: a randomized controlled trial”

	Study period				
	Enrolment	Allocation	Post-allocation		Close-out
Timepoint	Apr/2012–Dec/2016	Jan/2015–Jan/2017	Jan/2015–Jan/2017	Feb/2015–Feb/2017	Mar/2017
Enrolment:					
Eligibility screen	X				
Informed consent	X	X			
Allocation		X			
Interventions:					
Reduction mammaplasty		X			
[Antibiotic or placebo prescription]			X	X	
Assessments:					
[Eligibility criteria]	X	X			
[SSI assessment ]			-	X	X

### Data collection methods

Researchers will collect demographic data using a standardized form for each patient during their first medical appointment. Data from weekly postoperative appointments evaluating SSIs will also be collected using a standardized form.

### Data management

All data collected during the study will be entered into a unique spreadsheet, respecting the allocation and blinding. At the end of the study, after the last patient has been recruited, operated on and completed follow-up, the allocation group of each patient will be revealed and separate spreadsheets will be prepared for the data from experimental and placebo groups. The data from these two spreadsheets will be then compared with each other. Each patient will be identified only by a protocol number (the number of her package), to protect her privacy.

## Statistical methods

### Statistical methods – outcomes

The data will be entered into the statistical software Bioestat, version 5.0 (Instituto Mamirauá, Belém, Brazil). Descriptive statistics will be used to evaluate numerical variables, with calculation of the median, mean and standard deviation. The Mann-Whitney test will be used to compare the groups in age, BMI, duration of surgery, and weight of resected breast tissue.

The chi-square test or Fisher’s exact test will be used to compare the occurrence of SSIs among the groups. Those tests will be also used to study associations between the occurrence of infection and age, BMI, duration of the surgery, and weight of resected breast tissue in each group. For these variables, the values for each group will be divided into below the median and above the median.

### Statistical methods – analysis population and missing data

Data analysis will be performed based on the original allocation of all patients as defined by randomization, regardless of the degree of adherence to the protocol (intention-to-treat principle). With respect to missing data, the amount thereof, their patterns and the variables associated with omission will define the most appropriate technique to be used in the processing of such data, whether in the primary analysis or the sensitivity analysis. For primary analyses, methods will be used that make use of all available data, such as multiple imputations.

### Monitoring

In addition to the weekly evaluation regarding SSI, all the patients will be instructed to contact the assistant surgeon if they have any issue related to the operation or to the use of the capsules. In case of a diagnoses of SSI or if any problem related to the use of the capsules is recorded, the intake of the capsules will be immediately discontinued and the appropriate conduct will be taken in each case.

### Ethics and dissemination

The study protocol was approved by the institutional ethics committee, and all the subjects signed an informed consent form prior to enrollment.

### Protocol amendments

Any modifications of the protocol that may impact the study, the potential benefit to patients or patient safety, including changes of the study objectives, study design, patient population, sample size, study procedures or other significant aspects will require a formal amendment to the protocol. Such modification shall be agreed upon between the researchers and approval will be obtained from the Research Ethics Committee before implementation.

### Confidentiality

All participant information will be kept in password-protected files with limited access. Data that identify the participants will be replaced by codes.

### Access to data

All researchers involved in the study will have access to all data collected.

### Dissemination policy – authorship

The criteria for authorship of this protocol followed the guidelines established by the International Committee of Medical Journal Editors. A team of professional translators was used to translate the text from Portuguese into English. The final report will follow the main CONSORT 2010 guidelines.

## Discussion

Breast reduction was the eleventh most performed cosmetic procedure in the United States in 2014 (American Society of Plastic Surgeons). It is classified as a “clean operation”, although it has higher rates of infection than other procedures in the same category [18].

Studies comparing factors that affect the occurrence of SSIs in breast surgeries such as smoking, use of a surgical drain, preoperative chemotherapy, breast reconstruction, and associated administration of antibiotic prophylaxis, have presented conflicting conclusions [17, 29, 37, 43–47]. In three SSI cohort studies the recruited patients had differences in risk factors, preventing a quality meta-analysis [17, 43, 48, 49]. Finally, most of the available studies are related to the treatment of breast cancer and not specifically to breast reduction, emphasizing the importance of this study.

We chose to exclude women who have risk factors for SSIs in an attempt to homogenize the sample, allowing us to isolate the postoperative prophylaxis factor. In addition, pre- and intraoperative care related to infection control will be taken, including antiseptic treatment of the operative field, routine antibiotic prophylaxis, hemostasis, care and caution to detachments, handling and preparation of the flaps without tension sutures and sterile dressing will be taken.

Short et al. [18] performed a meta-analysis on SSIs from reduction mammaplasties, and found only three randomized clinical trials. They concluded that preoperative antibiotic prophylaxis is effective in preventing SSIs in reduction mammaplasties; they determined a decrease in the incidence of SSIs of 75%, compared with placebo or no medication, and therefore recommended its use in clinical practice. Their meta-analysis also highlighted the importance of randomized controlled trials to evaluate the postoperative prophylaxis, which has yet to be done [18, 23, 29, 50].

As well as in other studies in which cephalosporins are the main used antibiotics following reduction mammaplasties, we chose to use two different first-generation cephalosporins: cephalothin, for intravenous use in the intraoperative period and the first 24 hours (1 g each 6 hours) and cephalexin for oral use (500 g every 6 hours) in the subsequent postoperative 7 days, compared to placebo [9, 19, 21–23, 27–31].

The objective of the current study is to evaluate the role of antibiotic prophylaxis extended through the postoperative 7 days, in patients who have intraoperative use of cephalothin (every 6 hours, during the first 24 hours), seeking to assess the impact of extended prevention in the SSI rates.

Based on the evidence described and taking into account the study design, the results may justify standard protocols for the use of antimicrobial prophylaxis in breast reduction.

### Trial status

This trial is in the recruiting stage. The first patient was randomized in April 2012; by January 2016, a total of 90 patients had undergone surgery, and none of the patients had any infection during the first 30 days.

## Abbreviations

BMI: body mass index
CDC: Centers for Disease Control and Prevention
SSI: surgical site infection
